# Strengthening contraceptive uptake for migrants in border areas Tak Province, Thailand and internally displaced in Kayin State, Myanmar, 2020–2024^☆,☆☆^

**DOI:** 10.1016/j.contraception.2026.111423

**Published:** 2026-02-26

**Authors:** Nay Win Tun, Nan San Wai, Mary Ellen Gilder, Aye Kyi Win, Hsa Eh, Rose McGready

**Affiliations:** a Shoklo Malaria Research Unit, Mahidol-Oxford Tropical Medicine Research Unit in Faculty of Tropical Medicine, Mahidol University, Mae Sot, Thailand; b Centre for Tropical Medicine and Global Health in Nuffield Department of Medicine, University of Oxford, Oxford, UK

**Keywords:** Access, Internally Displaced, Long-Acting Reversible Contraception, Myanmar, Thailand, Migrants

## Abstract

**Objectives:**

We analyzed contraceptive utilization among rural migrant populations accessing fixed and outreach clinics under the Strengthening Migrant Access to Reproductive Healthcare in Tak Province, Thailand initiative (SMARH-T), and internally displaced at fixed clinics in Eastern Myanmar.

**Study Design:**

Observational, retrospective review of contraceptive utilization from 2020 to 2024 at 19 outreach clinics (all in Thailand) and four fixed clinics (two in Thailand, two in Myanmar). SMARH-T was launched in 2021 only in Thailand.

**Results:**

Consultations in conflict affected Myanmar fixed clinics was lower than in Thailand. Outreach clinics hosted the majority (54.6%) of the 20,959 consultations, mostly for short-acting hormonals (66.9%) and mainly depot medroxyprogesterone acetate (88.4%). Women chose implants (96%) at consultations involving long-acting reversible contraceptive methods (31.7%). Total consultations increased 4.2-fold from 2020 to 2024.

**Conclusions:**

Outreach clinics enhanced accessibility and significantly increased contraceptive uptake for marginalized populations. This model effectively expands reproductive healthcare in rural, border, resource-limited settings.

## 1 Introduction

Of the 800 daily maternal deaths worldwide in 2020, 95% occurred in low- and middle-income countries [1]. Contraceptives are a proven, life-saving intervention for women and girls of re-productive age, offering a critical pathway to reduce maternal mortality [2]. Access is the primary obstacle to contraceptive uptake for marginalized populations in rural or hard-to-reach areas [3].

An estimated 140,000 Myanmar nationals are based in Tak Province Thailand including refugees in camps and migrants (documented or undocumented) outside camps. In rural areas of the Thailand-Myanmar border contraceptive access barriers include: distance, finance, language and cultural differences in Thai clinics, and lack of legal documents [4,5]. A significant portion of the 260,000 (UNHCR 2026 estimates) internally displaced in Kayin State, Myanmar reside in temporary safe areas close to the Moei River which forms the border. This contributes to higher maternal mortality rates in Myanmar compared to Thailand [6]. To address this gap, Shoklo Malaria Research Unit (SMRU) launched the Strengthening Migrant Access to Reproductive Healthcare in Tak, Thailand (SMARH-T) initiative in late 2020. The program established 19 out-reach clinics in Thailand, to complement the four long-standing, fixed clinic structures that SMRU operates [4,5]. The fixed clinics, two in each country, provide 24-hour essential care including antenatal care, childbirth, neonatal care, contraceptive services, vaccination, and general out- and in-patient care. Outreach services are provided by two outreach teams that operate from the fixed clinics and conduct four visits per week, with most sites visited once per two weeks on the same day (e.g. Tuesday 7th and Tuesday 21st). Three sites with higher caseloads are visited once per week and three with lower caseloads once per month. Outreach visits provide antenatal care, contraception, vaccinations and unwell under-five years-old visits.

**Fig. 1:**
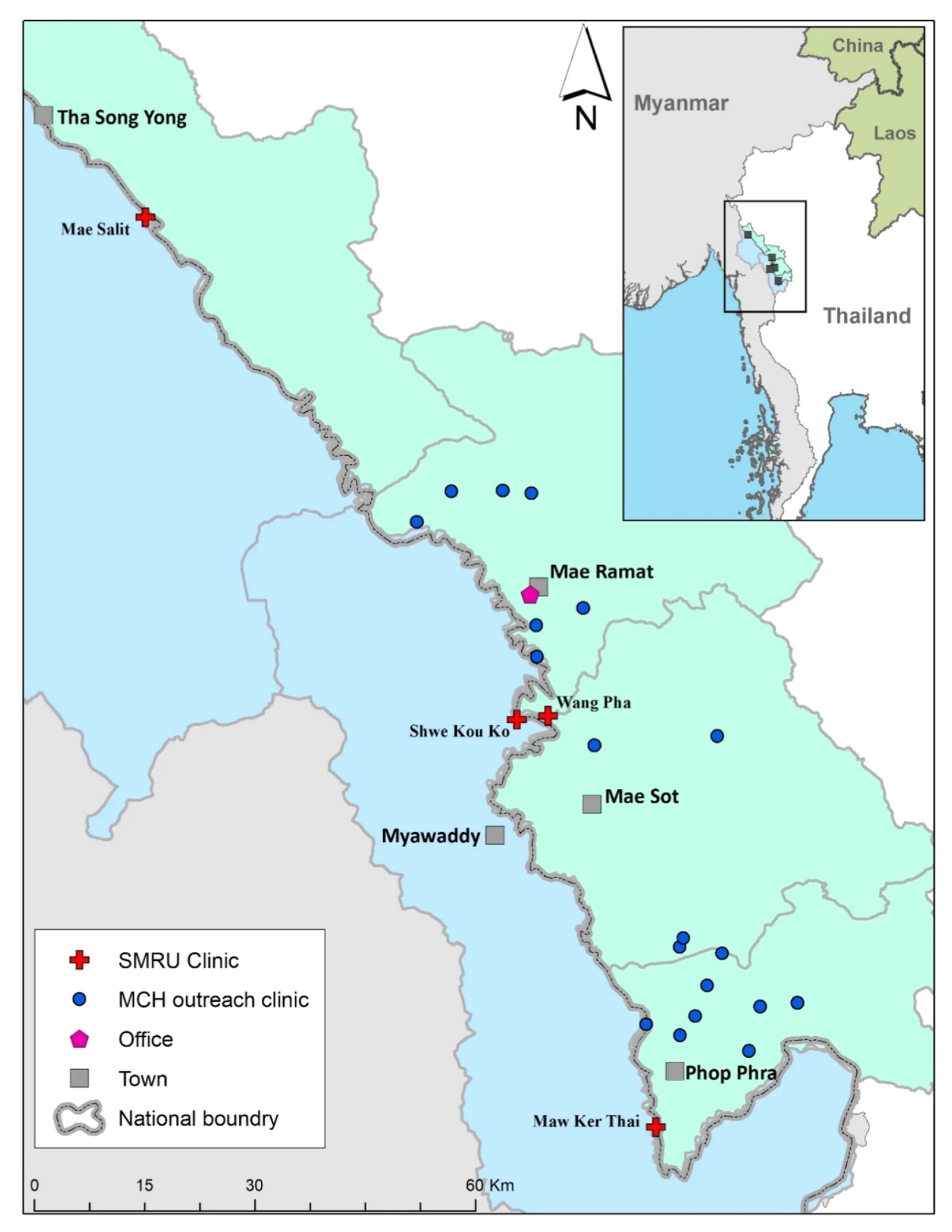
Map of the Shoklo Malaria Research Unit fixed and outreach clinics where contraceptives services are available for rural, marginalized migrant populations at the Thailand Myanmar border 2020–2024.

This analysis of rural, undocumented, migrants and internally displaced assesses contraceptive utilization including short-, long-acting reversible and permanent methods, across outreach clinics and fixed clinics in rural areas of Thailand and Myanmar (Fig. 1).

## 2 Materials and methods

### 2.1 Study design

We conducted an observational, retrospective review of anonymized electronic records, analyzing five years of contraceptive utilization in rural SMRU clinics in Tak Province, western Thailand and Kayin State, eastern Myanmar. We excluded consultations that did not result in contraceptive uptake. We used IBM SPSS v29.0 and summarized data as proportions.

### 2.2 Contraceptive availability

SMRU aims to provide a choice of modern, effective and free-of-charge methods: including short-acting (oral contraceptive pills, three month depot medroxyprogesterone acetate (DMPA)), long-acting reversible methods (implants, intrauterine devices (IUDs) and permanent methods (tubal ligation). Implant insertion occurred in both outreach and fixed clinics whereas IUD insertion was limited to fixed clinics.

### 2.3 Implant training

Between 2020 and 2024, we conducted four implant trainings including theory and practice. The six-hour training included 71 providers from migrant and minority language groups. Each participant required a minimum of three supervised insertions after successful completion of theoretical training.

### 2.4 Community health volunteer (CHV) training

The SMARH-T initiative objectives included strengthening capacity of CHVs to promote safe sexual and reproductive health (SRH) activities in the community. We provided a SRH curriculum for CHVs which included topics like: Counseling, Family Planning, and Adolescent health, to support this. Training was provided in Karen or Burmese language and completed by 121 CHVs between 2021–2022. In 2023–2024 these volunteers conducted more than 50 activity sessions within their own migrant clusters in Thailand, building trust and awareness surrounding contraceptive choices.

### 2.5 Ethics approval and consent to participate

The research is part of health system strengthening on SMARH-T for undocumented migrants in collaboration with Thai Public Health. Oxford University Tropical Research ethics committee: OxTREC reference number 537–23; and the local TAK community advisory board TCAB-202313, granted approval.

## 3 Results

### 3.1 Contraceptive uptake

Most contraceptive uptake was in Thailand 16,566/20,959 (79.0%) not Myanmar (Table 1). In Thailand, outreach clinics accounted for the majority of the consultations 9051/16,566 (54.6%). Between 2020 and 2024, the program achieved a 4.2-fold increase in volume over the five years, rising from 1624 consultations in 2020 to nearly 7000 in 2024 (Table 1).

### 3.2 Contraceptive methods

The majority of consultations were for short-acting contraceptives 14,027/20,959 (66.9%); mostly DMPA 12,394/14,027 (88.4%). Nearly one in three women, 6629/20,959 (31.7%) selected long-acting reversible contraception, mostly implants 6365/ 6629 (96.0%). Only a small proportion of women accessed permanent tubal ligation 303/20,959 (1.4%).

## 4 Discussion

The outreach model of care facilitated contraceptive uptake for undocumented migrants in rural border areas [4,5]. The SMARH-T initiative outreach clinics opened when COVID-19 movement restrictions on migrants peaked in Thailand [7,8]. Post COVID-19, outreach remains the main point of contraceptive uptake in contrast to Myanmar where escalating conflict restricts operations, leaving only two fixed facilities providing services as analyzed in this report. Contraceptives were included as part of the SMARH-T initiative package to reduce pregnancy related mortality among rural migrant women but the high contraceptive uptake suggests a previously unrecognized, unmet need. Strong demand for DMPA use aligns with previously published data from the Thai-Myanmar border [9]. IUD uptake was low despite earlier positive reports in the area [10], perhaps because it was less convenient than implant. Tubal ligation rates are low which differs from the high rates observed in Thai women [2].

Improving contraceptive accessibility in low-resource settings helps reduce existing SRH health inequalities: reducing unintended pregnancies, unsafe abortion, improving maternal and child health, and supporting economic development by enabling migrants to achieve better socio-economic stability [2]. Successful programs are possible when they address barriers, promote education, and enhance access to quality contraceptive services. This program produced long-term skill acquisition for minority health care workers and sustained community buy-in. Donor organizations were vital to this program as the cost of an implant (USD 64) is significant relative to the monthly wage (USD 64–160) of undocumented, agricultural migrants (if and when work is available).

In conclusion, the accessibility and availability of highly effective contraception led to high uptake among rural and undocumented migrants. Conflict hinders the delivery of sexual and reproductive health care services risking adverse reproductive health outcomes for women and children.

## Research data for this article

Data cannot be shared publicly because of the sensitivity of data for this population of undocumented migrants. De-identified participant data are available from the Mahidol Oxford Tropical Medicine Data Access Committee upon request from this link: https://www.tropmedres.ac/units/moru-bangkok/bioethics-engagement/datasharing.

## Figures and Tables

**Table 1:** Total contraceptive consultations (*n* = 20,959) at fixed and outreach clinics of Shoklo Malaria Research Unit for rural, marginalized populations, at the Thailand-Myanmar border 2020–2024

Total consultations	Frequency, *n* (%) (*n* = 20,959)
Clinic provision model	
Thailand (total consultations)	16,566 (79.0)
- Outreach clinics	9051 (43.2)
- Fixed clinics	7515 (35.8)
Myanmar (Kayin state)	
- Fixed clinics	4393 (21.0)
Year	
2020	1624 (7.7)
2021	2008 (9.6)
2022	4220 (20.1)
2023	6275 (30.0)
2024	6832 (32.6)
Type of contraceptive method	
Short-acting methods	14,027 (66.9)
- Depot medroxyprogesterone acetate	12,394 (59.1)
- Oral Contraceptive Pill	1633 (7.8)
Long-acting reversible contraception	6629 (31.7)
- Implant	6365 (30.4)
- Intrauterine devices	264 (1.3)
Permanent method (tubal ligation)	
- Tubal ligation	303 (1.4)
